# Causality between Telomere Length and the Risk of Hematologic Malignancies: A Bidirectional Mendelian Randomization Study

**DOI:** 10.1158/2767-9764.CRC-24-0402

**Published:** 2024-10-28

**Authors:** Guoyun Jiang, LingXiao Cao, Yunshan Wang, Li Li, Zie Wang, Hui Zhao, Yang Qiu, Bin Feng

**Affiliations:** 1 Department of Clinical Laboratory, Shandong Provincial Hospital Affiliated to Shandong First Medical University, Jinan, China.; 2 Department of Neurology, The Second Hospital of Shandong University, Cheeloo College of Medicine, Shandong University, Jinan, China.; 3 Department of Medical Oncology, Shandong Provincial Hospital Affiliated to Shandong First Medical University, Jinan, China.

## Abstract

Growing evidence indicates a relationship between telomere length (TL) and the stage, prognosis, and treatment responsiveness of hematopoietic malignancies. However, the relationship between TL and the risk of hematologic malignancies remains unclear, considering the vulnerability of observational studies to potential confounding and reverse causation. A two-sample bidirectional Mendelian randomization (MR) analysis was conducted utilizing publicly available genome-wide association study data to assess whether TL was causally associated with the risk of hematologic malignancies. The inverse variance weighted approach was used as the primary assessment approach to evaluate the effects of the causes, augmented by the weighted median and MR-Egger methods. Cochran’s Q test, MR-Egger intercept test, MR-Pleiotropy Residual Sum and Outlier test, and leave-one-out analysis were performed to evaluate sensitivity, heterogeneity, and pleiotropy. According to forward MR estimations, longer TL was related to an increased risk of acute lymphocytic leukemia (OR = 2.690; *P* = 0.041), chronic lymphocytic leukemia (OR = 2.155; *P* = 0.005), multiple myeloma (OR = 1.845; *P* = 0.024), Hodgkin lymphoma (OR = 1.697; *P* = 0.014), and non–Hodgkin lymphoma (OR = 1.737; *P* = 0.009). Specific types of non–Hodgkin lymphoma were also associated with TL. The reverse MR results revealed that hematologic malignancies had no effect on TL. This MR analysis revealed an association between longer TL and an increased risk of specific hematologic malignancies, indicating a potential role of TL in risk evaluation and management in hematologic malignancies.

**Significance:**

In contrast to observational studies, this study uncovered the reliable causal relationships between TL and hematologic malignancies, emphasizing the potential role of telomeres in tumor development. TL maintenance may offer a promising strategy to reduce the risk of hematologic malignancies.

## Introduction

Hematologic malignancies are among the most prevalent and rapidly developing malignant tumors, posing an enormous threat to human health (1). However, the development of effective prediction, treatment, and prevention strategies for hematologic malignancies encounter considerable challenges, primarily due to their unclear nature of pathogenesis. Although research has indicated that radiation (1), smoking (2), and chemical carcinogens (3) may increase the risk of developing hematologic malignancies, the exact cause of hematologic malignancies remains unclear, and there is no reliable approach for predicting hematologic malignancies.

Telomeres are unique DNA–protein complexes located at the ends of chromosomes, consisting of a G-rich DNA sequence with associated proteins. Human telomeres are typically composed of TTAGGG repeats, and their primary function is to prevent chromosomal DNA degradation, terminal fusion loss, and aberrant recombination, thus maintaining chromosome stability and integrity (4). Telomeres progressively shorten with cellular division in normal human cells, limiting their lifespan and serving as a critical barrier against excessive proliferation and malignant transformation. In contrast, telomere maintenance is a hallmark of human malignant cells and is necessary for their limitless proliferation (5). Multiple investigations suggest that telomere length (TL) may be important in the development and progression of cancer, particularly hematologic malignancies (6). According to research, TL in acute myeloid leukemia (AML; ref. 7), chronic myeloid leukemia (CML; refs. 8, 9), non–Hodgkin lymphoma (NHL; ref. 10), and other hematologic malignancies is longer than TL in normal blood cells. However, the causal associations between TL and hematologic malignancies remain controversial, and various observational studies have yielded conflicting data about the impact of TL on malignancy. Shorter TL has been shown in several studies to be a probable predictor of AML (11, 12), CML (13), acute lymphoblastic leukemia (ALL; refs. 14, 15), chronic lymphoblastic leukemia (CLL; refs. 16, 17), and NHL (18). The inadequacy of the research methodologies utilized is one plausible explanation for the emergence of these conflicting results. The question of whether TL alteration is a cause or a consequence of the development of hematologic malignancies remains unanswered.

Mendelian randomization (MR) is an instrumental variable (IV) method that uses single-nucleotide polymorphisms (SNP) as IVs to infer causal links between two traits, with the advantages of minimizing confounders and bias for reverse causation (19). The MR study provides an evaluation of causality that can aid in the prevention of divergence caused by confounding factors. MR studies have been widely used to investigate the causal relationships between potential risk factors and disease risk, such as TL and cancer (20), body mass index and diabetes (21), smoking and stroke (22), and so forth. However, the bidirectional causal association between TL and hematologic malignancies has yet to be thoroughly elucidated. In this study, we used a bidirectional two-sample MR method, leveraging its unique advantages, to investigate the potential bidirectional association between TL and hematologic malignancies, aiming to provide evidence about the role of telomeres in potential targets for prediction, prevention, and treatment in hematopoietic malignancies.

## Materials and Methods

### Study design

**Figure 1:**
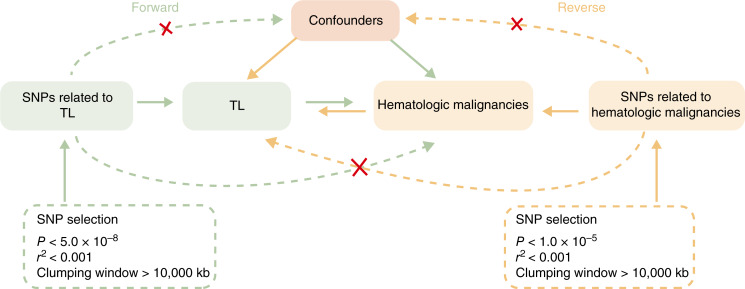
Principle diagram of MR analysis of the bidirectional causal relationship between TL and hematologic malignancies. In the forward MR analysis, TL was chosen as the exposure, with hematologic malignancies serving as the outcome. In the reverse MR analysis, hematologic malignancies were chosen as the exposure, with TL serving as the outcome.

The bidirectional MR analysis between TL and hematologic malignancies followed the “STROBE-MR” protocol, using the approach depicted in Fig. 1. Forward and reverse MR analyses were performed to investigate the bidirectional causal relationships between TL and hematologic malignancies. Based on summary statistics from the genome-wide association study (GWAS), SNPs associated with exposure were identified and utilized as IVs to assess causality with the outcome. In the forward MR analysis, TL was chosen as the exposure, with hematologic malignancies serving as the outcome. Conversely, in the reverse MR analysis, hematologic malignancies were chosen as the exposure, with TL serving as the outcome. This MR analysis was conducted based on the fundamental hypotheses that underlie MR: (i) IVs of interest exposures are highly correlated with exposures; (ii) IVs of interest exposures are irrelevant to confounders of interest exposure–outcome associations; and (iii) IVs only affect outcomes by influencing the interest exposure.

### Datasets and selection of IVs

We obtained summary statistics of TL from GWAS data on European ancestry from the Integrative Epidemiology Unit (IEU) OpenGWAS Project (ID: ieu-b-4879; Supplementary Table S1) consisting of 472,174 individuals. The GWAS information for primary lymphoid and hematopoietic malignant neoplasms, eight hematologic malignancies (AML, ALL, CML, CLL, multiple myeloma, Hodgkin lymphoma, NHL, and leukemia of unspecified cell type), and eight classifications of NHL (follicular lymphoma, nonfollicular lymphoma, diffuse large B-cell lymphoma, Waldenström macroglobulinemia/lymphoplasmacytic lymphoma, mantle cell lymphoma, marginal zone B-cell lymphoma, mature T/NK lymphomas, and other and unspecified types of NHL) were obtained from the FinnGen GWAS database (23).The GWAS cohorts exclusively consist of individuals of European ancestry (Supplementary Table S1), and the criterion of the participants can be found on FinnGen (www.finngen.fi/en). To further validate the effectiveness of the results across different populations, we additionally obtained GWAS data from the UK Biobank on IEU OpenGWAS Project (Supplementary Table S1), encompassing GWAS data of leukemia, lymphoid leukemia, and myeloid leukemia on European ancestry.

For IVs representing exposure, SNPs that passed the genome-wide significance threshold were subsequently clumped with other related SNPs within 10,000 kb based on the 1000 Genomes Project’s evaluation of linkage disequilibrium using *r*^2^ < 0.001. In the forward MR analysis, the TL significance threshold was selected at 5 × 10^−8^, and the IVs that were significantly associated with the outcome (*P* < 5 × 10^−8^) were excluded. In the reverse MR analysis, we adjusted the significance threshold to 1 × 10^−5^ in order to prevent unreliable outcomes caused by an absence of SNPs because there were limited IVs for hematopoietic malignancies (Fig. 1). Subsequently, we harmonized the exposure and outcome data to eliminate strand mismatches and guarantee the alignment of SNP effect sizes. F-statistics were used to estimate the strength of IVs. F > 10 indicated strong instrument strength, and IVs with F < 10 were excluded (24). The PhenoScanner v2 (http://www.phenoscanner.medschl.cam.ac.uk/) was used to exclude variations related to phenotypes that might be associated with hematologic malignancies in the forward MR analysis, as well as those associated with TL in the reverse MR analysis (25, 26).

### Statistical analysis

To evaluate the potential bidirectional causal connections between TL and hematologic malignancies, we conducted a two-sample bidirectional MR analysis using the inverse variance weighted (IVW) method, which is most commonly used in MR studies and offers robust causal estimates in the absence of directional pleiotropy. Additionally, the MR-Egger and weighted median (WM) methods were used for verifying the results. The MR-Egger regression was used for estimating the level of bias induced by directional pleiotropy. To detect and exclude outliers, the Mendelian Randomization Pleiotropy Residual Sum and Outlier (MR-PRESSO) and leave-one-out analyses were used. The heterogeneity was estimated by IVW and MR-Egger methods via Cochran’s Q statistics and funnel plots. The OR and 95% confidence interval (CI) were utilized to estimate the causal relationship between TL and hematologic malignancies. R version 4.3.1 and its package “TwoSampleMR (version 0.6.4)” were used for statistical analysis.

### Data availability

The datasets analyzed during the current study are available in the FinnGen (www.finngen.fi/en) and IEU OpenGWAS (gwas.mrcieu.ac.uk/) databases.

## Results

### The effect of TL on hematologic malignancies

**Figure 2:**
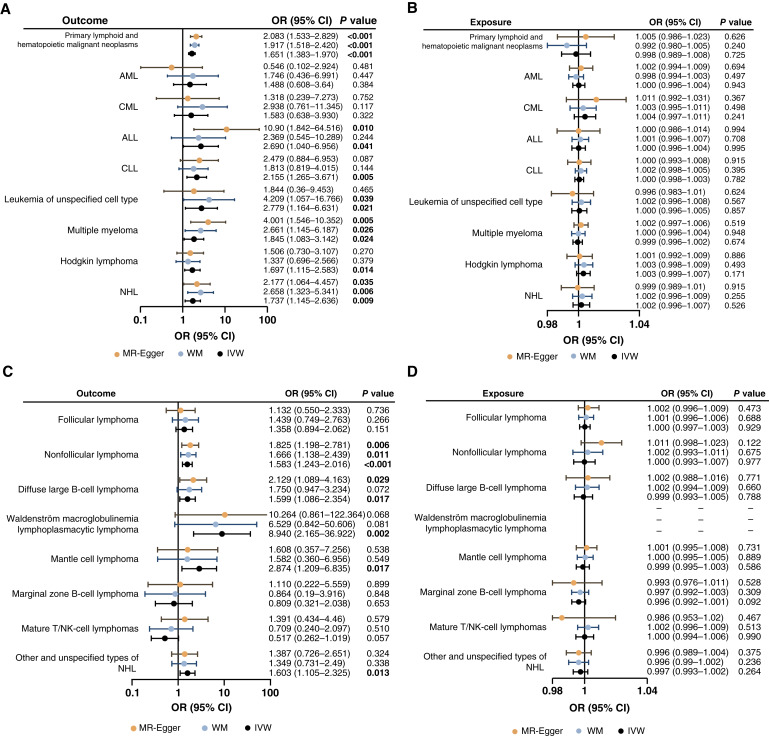
Forest plots showing the forward and reverse causal associations between TL and hematopoietic malignancies. The dots in this forest plot show the value of OR, and the horizontal lines demonstrate the range of 95% CI. **A,** Causal association between TL and different types of hematopoietic malignancies. **B,** Reverse causal association between TL and different types of hematopoietic malignancies. **C,** Causal association between TL and different types of NHL. **D,** Reverse causal association between TL and different types of NHL.

As shown in Fig. 2A and Table 1, the result of the IVW analysis demonstrated that TL was significantly associated with primary lymphoid and hematopoietic malignant neoplasms (OR = 1.650; 95% CI, 1.383–1.970; *P* = 2.69 × 10^−8^). In detail, IVW results indicated that TL was associated with leukemia of unspecified cell type (OR = 2.779; 95% CI, 1.164–6.631; *P* = 0.021) and lymphoid hematologic malignancies, including ALL (OR = 2.690; 95% CI, 1.040–6.956; *P* = 0.041), CLL (OR = 2.155; 95% CI, 1.265–3.671; *P* = 0.005), multiple myeloma (OR = 1.845; 95% CI, 1.083–3.142; *P* = 0.024), Hodgkin lymphoma (OR = 1.697; 95% CI, 1.115–2.583; *P* = 0.014), and NHL (OR = 1.737; 95% CI, 1.145–2.636; *P* = 0.009; Fig. 2A; Table 1; Supplementary Fig. S1). The results of the MR-Egger and WM methods displayed consistency (OR > 1), subsequently reinforcing the reliability of the analysis. On contrast, TL was not significantly associated with myeloid leukemia, including AML (OR = 1.488; 95% CI, 0.608–3.640; *P* = 0.384) and CML (OR = 1.583; 95% CI, 0.638–3.930; *P* = 0.322), as confirmed by MR-Egger and WM methods. As a result, longer TL was predicted to be related to an increased risk of primary lymphoid and hematopoietic malignant neoplasms, including lymphoid hematologic malignancies and leukemia of unspecified cell type.

**Table 1:** The forward MR analysis on TL and hematologic malignancies

Outcome	nSNP	Methods	*P* value^∗^	OR	CI (95%)	*P* (*heterogeneity*)		*P* (*pleiotropy*)	MR-PRESSO
						MR-Egger	IVW		
Primary lymphoid and hematopoietic malignant neoplasms	116	MR-Egger	**7.53E−6**	2.083	1.533–2.829	0.000	0.000	0.073	0.008
		WM	**4.61E**−**8**	1.917	1.518–2.420				
		IVW	**2.69E−8**	1.651	1.383–1.970				
AML	112	MR-Egger	0.481	0.546	0.102–2.924	0.330	0.307	0.170	0.297
		WM	0.447	1.746	0.436–6.991				
		IVW	0.384	1.488	0.608–3.640				
CML	115	MR-Egger	0.752	1.318	0.239–7.273	0.096	0.106	0.804	0.112
		WM	0.117	2.938	0.761–11.345				
		IVW	0.322	1.583	0.638–3.930				
ALL	115	MR-Egger	**0.010**	10.901	1.842–64.516	0.748	0.692	0.071	0.713
		WM	0.244	2.369	0.545–10.289				
		IVW	**0.041**	2.690	1.040–6.956				
CLL	115	MR-Egger	0.087	2.479	0.884–6.953	0.943	0.950	0.756	0.956
		WM	0.144	1.813	0.819–4.015				
		IVW	**0.005**	2.155	1.265–3.671				
Leukemia of unspecified cell type	114	MR-Egger	0.465	1.844	0.360–9.453	0.879	0.887	0.562	0.882
		WM	**0.039**	4.209	1.057–16.766				
		IVW	**0.021**	2.779	1.164–6.631				
Multiple myeloma	117	MR-Egger	**0.005**	4.001	1.546–10.352	0.252	0.198	0.058	0.191
		WM	**0.026**	2.661	1.145–6.187				
		IVW	**0.024**	1.845	1.083–3.142				
Hodgkin lymphoma	118	MR-Egger	0.270	1.506	0.730–3.107	0.316	0.336	0.691	0.344
		WM	0.379	1.337	0.696–2.566				
		IVW	**0.014**	1.697	1.115–2.583				
NHL	117	MR-Egger	**0.035**	2.177	1.064–4.457	0.058	0.061	0.449	0.058
		WM	**0.006**	2.658	1.323–5.341				
		IVW	**0.009**	1.737	1.145–2.636				

^∗^Bold values indicate significant results.

The Cochran’s Q test, applied in conjunction with the IVW and MR-Egger methods, indicated that there was no heterogeneity in the IVs in the eight hematologic malignancies (*P* > 0.05), whereas there was heterogeneity in the primary lymphoid and hematopoietic malignant neoplasms (*P* < 0.05). Furthermore, the leave-one-out analysis demonstrated that the results remained stable regardless of the removal of any single SNP (Supplementary Fig. S2). As depicted in the funnel plot (Supplementary Fig. S3), the symmetrical distribution of effect size variations around the point estimate suggested that there was no discernible evidence of horizontal pleiotropy. Moreover, the MR-PRESSO analysis did not detect any potential instrumental outliers at the nominal significance level of 0.05 in the eight hematologic malignancies. Using the MR-Egger regression intercept method, we found no evidence of horizontal pleiotropy for TL in hematologic malignancies with *P* > 0.05.

### The effect of hematologic malignancies on TL

The reverse causality bias may exist due to the potential impact on TL in hematologic malignancies and the possibility that an IV causes the outcome which in turn causes the exposure (27). Consequently, a reverse MR analysis was performed to exclude the possibility of reverse causality bias. The IVW analysis indicated no significant causal association between hematologic malignancies and TL (*P* > 0.05). The MR-Egger and WM methods were used as well, and the results concurred with the IVW analysis (Fig. 2B; Supplementary Fig. S4; Supplementary Table S2).

The MR-Egger regression, MR-PRESSO, and funnel plot analyses revealed no possible pleiotropy (*P* > 0.05). The Cochran’s Q test of IVW and MR-Egger methods confirmed no heterogeneity (*P* > 0.05) in the IVs (Supplementary Table S2). In conclusion, these results demonstrated that the causal relationship between TL and hematologic malignancies was stable and reliable, with no significant confounders and bias.

### The associations between NHL and TL

In the preceding analysis, we discoursed a casual effect of TL on NHL, and we further investigated the relevance of TL for the classification of NHL. NHL accounts for 85% of all lymphoma cases and has several subtypes, including follicular lymphoma, nonfollicular lymphoma, diffuse large B-cell lymphoma, lymphoplasmacytic lymphoma (Waldenström macroglobulinemia), mantle cell lymphoma, marginal zone B-cell lymphoma, mature T/NK lymphomas, and other unspecified types. The effect of TL on the risk of developing each kind of lymphoma was further investigated. The results of the forward MR analysis suggested an association between TL and the risk of nonfollicular lymphoma (*P* = 1.98 × 10^−4^), diffuse large B-cell lymphoma (*P* = 0.017), lymphoplasmacytic lymphoma (Waldenström macroglobulinemia; *P* = 0.002), mantle cell lymphoma (*P* = 0.017), and other and unspecified types of NHL (*P* = 0.013; Fig. 2C; Table 2; Supplementary Fig. S5). The results indicated that longer TL was predicted to be associated with an elevated risk of developing these types of NHL. However, no significant association was observed between TL and the risk of follicular lymphoma (*P* = 0.151), marginal zone B-cell lymphoma (*P* = 0.653), or mature T/NK-cell lymphoma (*P* = 0.057).

**Table 2:** The forward MR analysis on TL and the classification NHL

Outcome	nSNP	Methods	*P* value^∗^	OR	CI (95%)	*P* (*heterogeneity*)		*P* (*pleiotropy*)	MR-PRESSO
						MR-Egger	IVW		
Follicular lymphoma	108	MR-Egger	0.736	1.132	0.550–2.333	0.068	0.073	0.547	0.083
		WM	0.266	1.439	0.749–2.763				
		IVW	0.151	1.358	0.894–2.062				
Nonfollicular lymphoma	110	MR-Egger	**0.006**	1.825	1.198–2.781	0.730	0.737	0.419	0.722
		WM	**0.011**	1.666	1.138–2.439				
		IVW	**1.98E−4**	1.583	1.243–2.016				
Diffuse large B-cell lymphoma	109	MR-Egger	**0.029**	2.129	1.089–4.163	0.791	0.789	0.309	0.807
		WM	0.072	1.750	0.947–3.234				
		IVW	**0.017**	1.599	1.086–2.354				
Waldenström macroglobulinemia, lymphoplasmacytic lymphoma	110	MR-Egger	0.068	10.264	0.861–122.364	0.068	0.077	0.894	0.075
		WM	0.081	6.529	0.842–50.606				
		IVW	**0.002**	8.940	2.165–36.922				
Mantle cell lymphoma	110	MR-Egger	0.538	1.608	0.357–7.256				0.743
		WM	0.549	1.582	0.360–6.956	0.740	0.743	0.358	
		IVW	**0.017**	2.874	1.209–6.835				
Marginal zone B-cell lymphoma	109	MR-Egger	0.899	1.110	0.222–5.559	0.227	0.243	0.639	0.250
		WM	0.848	0.864	0.190–3.916				
		IVW	0.653	0.809	0.321–2.038				
Mature T/NK-cell lymphomas	110	MR-Egger	0.579	1.391	0.434–4.460				0.379
		WM	0.510	0.709	0.240–2.097	0.465	0.382	0.044	
		IVW	0.057	0.517	0.262–1.019				
Other and unspecified types of NHL	110	MR-Egger	0.324	1.387	0.726–2.651	0.907	0.915	0.594	0.915
		WM	0.338	1.349	0.731–2.490				
		IVW	**0.013**	1.603	1.105–2.325				

^∗^Bold values indicate significant results.

We performed a MR reverse analysis to exclude the possibility of reverse causation. The findings indicated that the onset of follicular lymphoma, nonfollicular lymphoma, diffuse large B-cell lymphoma, mantle cell lymphoma, marginal zone B-cell lymphoma, mature T/NK-cell lymphomas, and other and unspecified types of NHL did not lead to changes in TL (Fig. 2D; Supplementary Fig. S6; Supplementary Table S3). However, an exception was observed in Waldenström macroglobulinemia (lymphoplasmacytic lymphoma), which yielded no results due to the absence of SNPs (Supplementary Table S3).

The results of the leave-one-out analysis exhibited stability (Supplementary Fig. S7), and upon investigation of heterogeneity and pleiotropy, the results conclusively demonstrated that there was no evidence of either heterogeneity or horizontal pleiotropy in both the forward and reverse MR analyses (Supplementary Fig. S8; Supplementary Table S3; *P* > 0.05).

### Replicated validation analysis

To further validate the effectiveness of the results in this study across different populations and datasets, we conducted MR analysis using GWAS data from the UK Biobank. The results showed that TL was significantly associated with leukemia (OR = 1.003; *P* = 2.35 × 10^−6^) and lymphoid leukemia (OR = 1.002; *P* = 1.45 × 10^−8^) but not associated with myeloid leukemia (Supplementary Table S4; *P* = 0.316), which aligns with the results above.

## Discussion

The relationship between TL and cancer risk has been the focus of various arguments in epidemiologic research, with inconsistent findings across cancer types (28). In this study, we conducted a MR analysis to investigate the association between TL and hematologic malignancies, ultimately uncovering a correlation between longer TL and an increased risk of certain hematologic malignancies.

TL plays a crucial role in maintaining genomic integrity and preventing the onset of cellular senescence or apoptosis. Numerous studies have suggested that TL may serve as a potential predictor of risk for hematologic malignancies. Studies have discovered that genetic variants associated with longer TL increase the risk of developing AML, CML, CLL, and NHL (7–10). However, some studies have contrasting results, indicating that individuals with shorter TL may have a higher risk of AML, ALL, CML, CLL, NHL, and other hematologic malignancies (11–13, 16–18).The reason for the contrasting results may be that traditional observational studies could be influenced by residual confounding, as they may be prone to bias from reverse causality and influenced by TL-related demographic and lifestyle factors. Additionally, studies have indicated that TL may undergo modifications, such as lengthening or shortening, following the onset of hematologic malignancies (29, 30). Consequently, retrospective studies face further limitations due to bias, as TL measurements are often conducted after diagnosis and/or treatment.

To address these concerns, we used the MR analysis approach, which differs significantly from observational studies. Our MR analysis enabled us to study the causal relationship between TL and hematologic malignancies, and the results were less prone to confounding factors and reverse causation than traditional observational methods. Similarly, two MR analyses independently corroborated a significant association between longer TL and an increased risk of hematopoietic malignancies (31, 32). Our research expands on this by investigating a broader spectrum of hematopoietic malignancies, using bidirectional MR analysis to eliminate potential causal reverse relationships, and subsequently validating these findings in an additional population. In this study, we found that TL was associated exclusively with lymphoid and hematopoietic malignant neoplasms, solely in the context of lymphatic system cancers, including ALL, CLL, Hodgkin lymphoma, and NHL (nonfollicular lymphoma, diffuse large B-cell lymphoma, lymphoplasmacytic lymphoma, and mantle cell lymphoma). The utilization of diverse MR methods, along with the detection of heterogeneity and pleiotropy, and replicated validations ensures the reliability of the results. However, there was heterogeneity in primary lymphoid and hematopoietic malignant neoplasms, potentially attributed to their encompassing of a wide spectrum of hematologic malignancies.

TL is a complex trait that is influenced by an intricate interplay of individual genetics, age, gender, epigenetics, environmental exposures, and a myriad of uncertain interacting factors (33). As cells divide, telomeres progressively shorten, and once a critical threshold is reached, they often trigger cellular senescence or apoptosis. During carcinogenesis, cells adopt pathways that enhance telomerase expression through telomerase reverse transcriptase (TERT) and/or utilize the alternative lengthening of telomeres (ALT) pathway to extend telomeres and continue dividing (34). A larger clonal expansion due to long TL would increase the risk of acquiring mutations and ultimately cause malignant transformation (28). Multiple genetic and epigenetic mechanisms, including amplifications, structural variants, promoter mutations, and promoter methylation (epigenetic modification), have been shown to induce TERT expression (33). ALT functions independently of telomerase and relies somewhat on homologous recombination (35). Disruptions in the regulation of TERT and ALT can lead to variations in TL among individuals, thereby increasing the risk of mutations and malignant transformation in hematopoietic malignancies.

We observed a significant correlation between TL and hematologic malignancies, which helped us grasp the malignancy–TL association. Our findings indicate that TL is linked to the risk of specific hematologic malignancies, suggesting its potential as a risk prediction tool or as a target for disease prevention interventions. Moreover, some studies indicate that lifestyle characteristics such as cigarette smoking (36, 37), diet (38, 39), physical activity (40, 41), and body mass index (42) are correlated with TL, which encourages people to adopt healthier lifestyles. TL maintenance may serve as a promising strategy to reduce the risk of hematologic malignancies through lifestyle modifications or targeted TL management. Furthermore, targeting telomere maintenance represents a compelling opportunity for malignancy treatment, though further investigation is required (43).

However, there were some limitations in this study. First, the genetic relationship between TL and the risk of developing hematologic malignancies was investigated in individuals of European descent, thereby potentially limiting its generalizability to other ethnicities. Second, the sample size of certain hematologic malignancies in the FinnGen database was relatively small, and the subtypes of leukemia in the UK Biobank have not been further differentiated. Consequently, the findings should be further validated in larger, ethnically diverse cohorts to ensure their generalizability and reliability. Last, the approach we used relies on the three fundamental assumptions underlying MR analysis, and failures of these hypotheses may distort the degree to which the association between genetic longitude and sickness is established.

In conclusion, we found that longer TL was associated with an increased risk of specific hematologic malignancies, emphasizing the crucial role of telomeres in tumor development. Consequently, individuals with elongated TL should maintain heightened awareness of their susceptibility to lymphatic system cancers. The precise role and underlying mechanisms of telomere in the risk of hematologic malignancies, as well as the efficacy of telomere maintenance in preventing and treating these malignancies, are crucial areas that require further investigation.

## Supplementary Material

Supplemental Figure 1 Supplemental Figure 1. Scatter plot of single nucleotide polymorphism potential effects on TL and hematopoietic malignancies.

Supplemental Figure 2 Supplemental Figure 2. Forest plot of the results of the leave-one-out analysis for forward MR analysis between TL and hematologic malignancies.

Supplemental Figure 3 Supplemental Figure 3. Funnel plot for TL displays the estimation obtained through the utilization of the inverse of the standard error of the causal estimate, with each individual SNP serving as a tool.

Supplemental Figure 4 Supplemental Figure 4. Scatter plot of single nucleotide polymorphism potential effects on hematopoietic malignancies and TL.

Supplemental Figure 5 Supplemental Figure 5. Scatter plot of single nucleotide polymorphism potential effects on TL and non-Hodgkin lymphoma.

Supplemental Figure 6 Supplemental Figure 6. Scatter plot of single nucleotide polymorphism potential effects on non-Hodgkin lymphoma and TL.

Supplemental Figure 7 Supplemental Figure 7. Forest plot of the results of the leave-one-out analysis for forward MR analysis between TL and non-Hodgkin lymphoma.

Supplemental Figure 8 Supplemental Figure 8. Funnel plot for TL displays the estimation obtained through the utilization of the inverse of the standard error of the causal estimate, with each individual SNP serving as a tool.

Supplemental Table 1 Supplemental Table 1. GWAS data information of TL and hematologic malignancies

Supplemental Table 2 Supplemental Table 2. The reverse MR analysis on telomere length and hematologic malignancies

Supplemental Table 3 Supplemental Table 3. The reverse MR analysis on telomere length and the classification non-Hodgkin lymphoma

Supplemental Table 4 Supplemental Table 4. The Replicated MR analysis on telomere length and hematologic malignancies

Supplemental Figure Legend Supplemental Figure Legend
