# Supplementary figures and images for: Causality between Telomere Length and the Risk of Hematologic Malignancies: A Bidirectional Mendelian Randomization Study
Source: Cancer Res Commun. 2024 Oct 28;4(10):2815–22. doi: 10.1158/2767-9764.CRC-24-0402 (PMC11513617; doi:10.1158/2767-9764.CRC-24-0402)

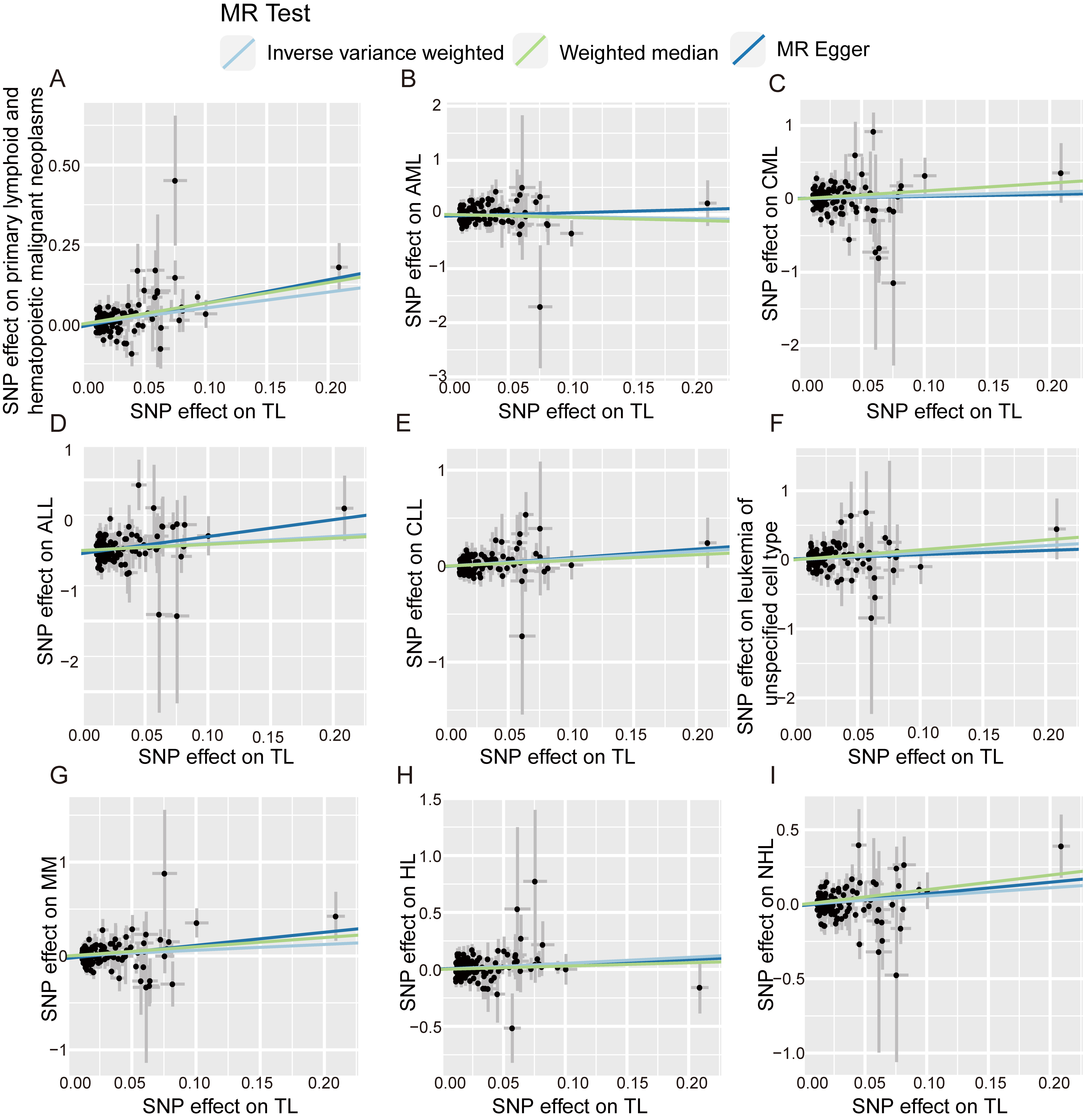

Supplement: Supplemental Figure 1 — Scatter plot of single nucleotide polymorphism potential effects on TL and hematopoietic malignancies. [file crc-24-0402_supplemental_figure_1_suppsf1.jpeg]

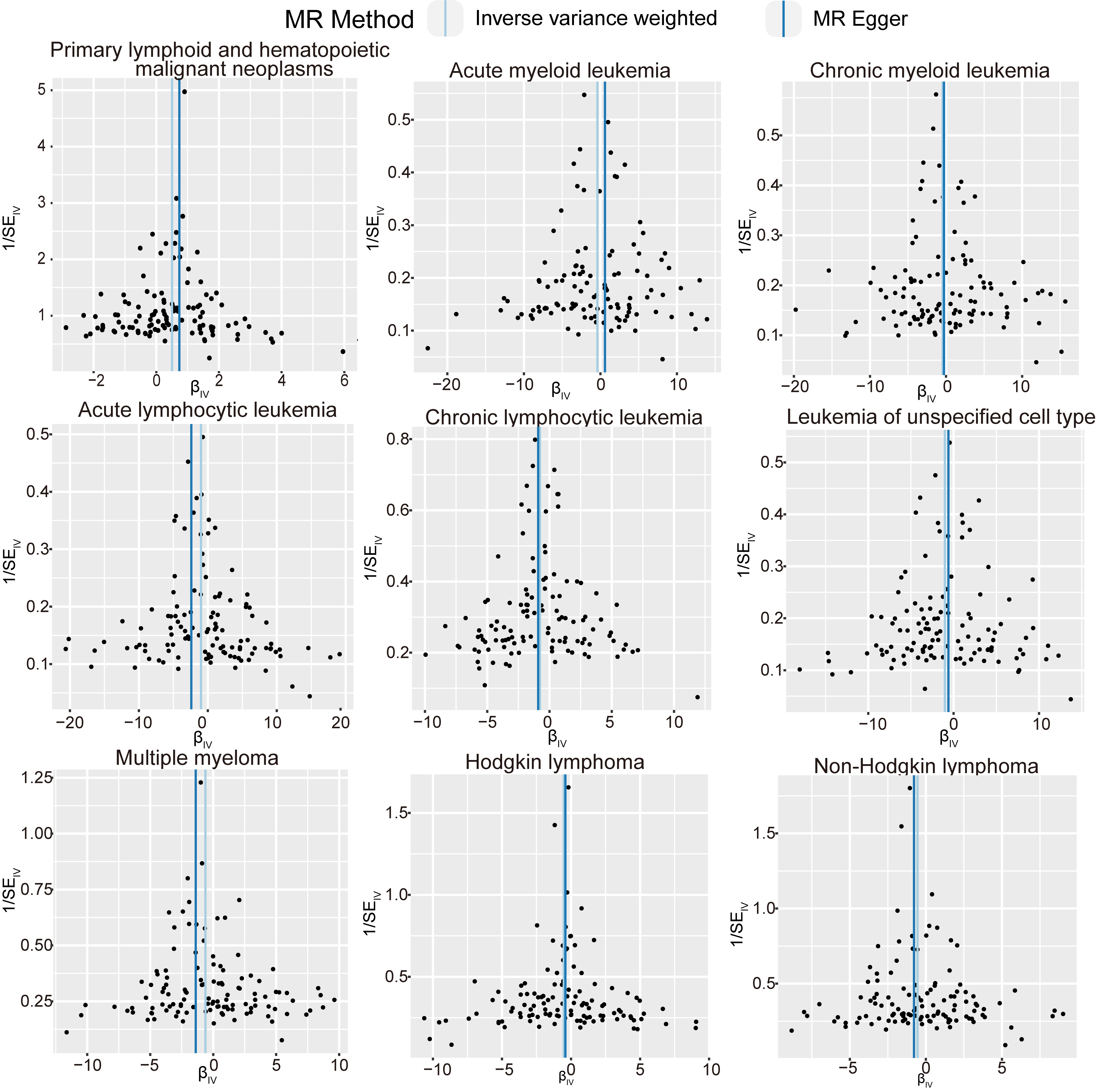

Supplement: Supplemental Figure 3 — Funnel plot for TL displays the estimation obtained through the utilization of the inverse of the standard error of the causal estimate, with each individual SNP serving as a tool. [file crc-24-0402_supplemental_figure_3_suppsf3.jpeg]

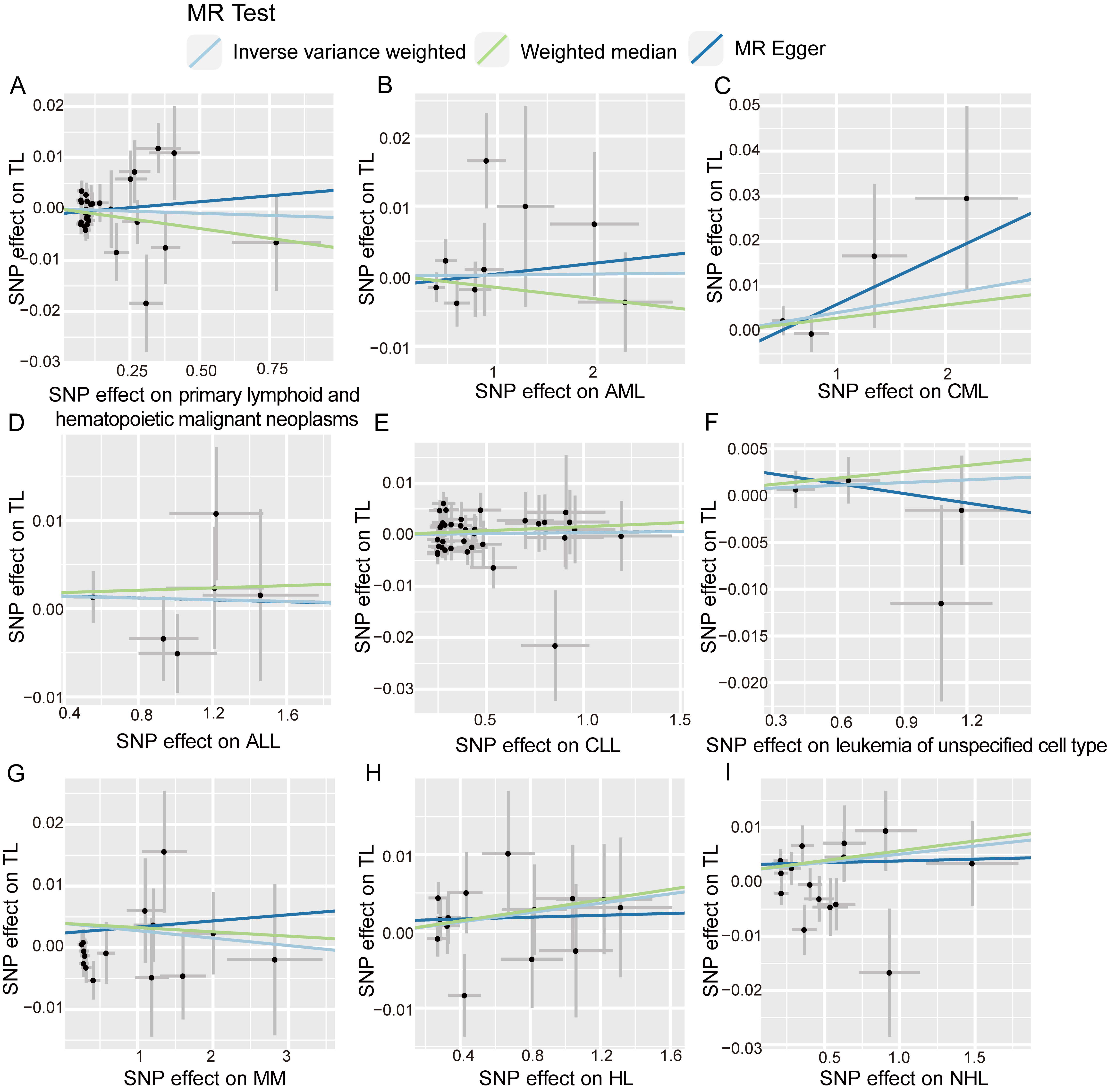

Supplement: Supplemental Figure 4 — Scatter plot of single nucleotide polymorphism potential effects on hematopoietic malignancies and TL. [file crc-24-0402_supplemental_figure_4_suppsf4.jpeg]

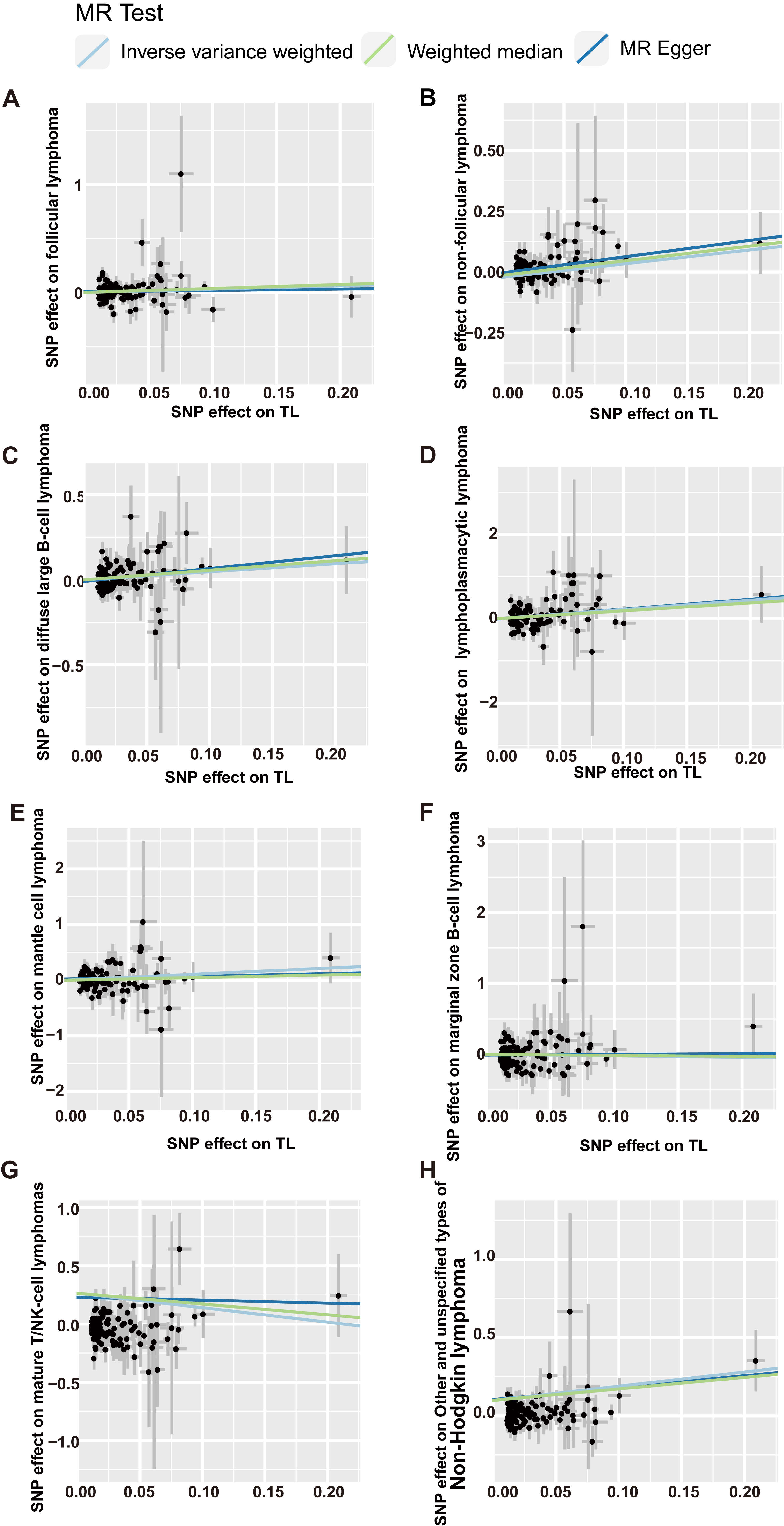

Supplement: Supplemental Figure 5 — Scatter plot of single nucleotide polymorphism potential effects on TL and non-Hodgkin lymphoma. [file crc-24-0402_supplemental_figure_5_suppsf5.jpeg]

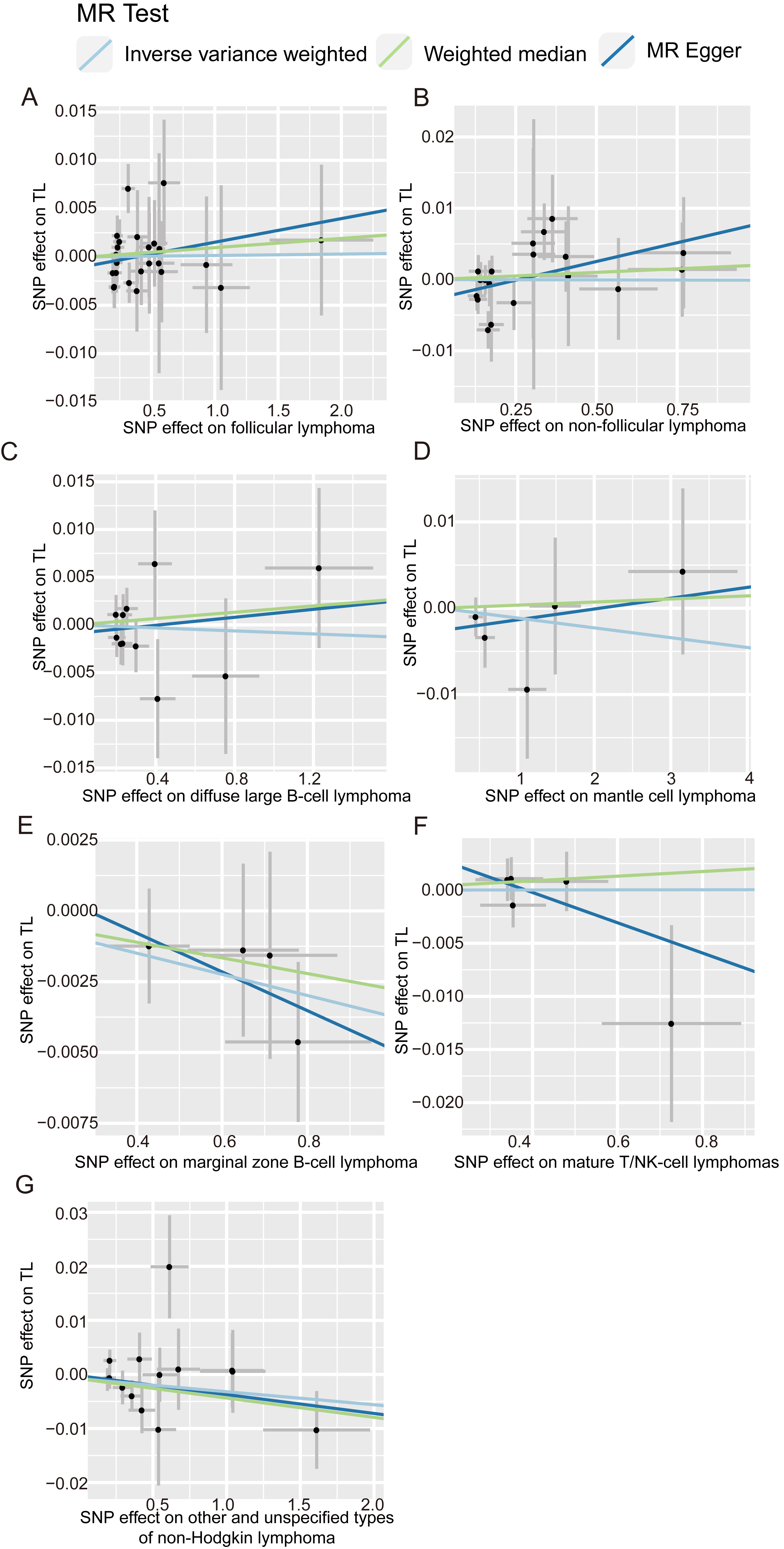

Supplement: Supplemental Figure 6 — Scatter plot of single nucleotide polymorphism potential effects on non-Hodgkin lymphoma and TL. [file crc-24-0402_supplemental_figure_6_suppsf6.jpeg]

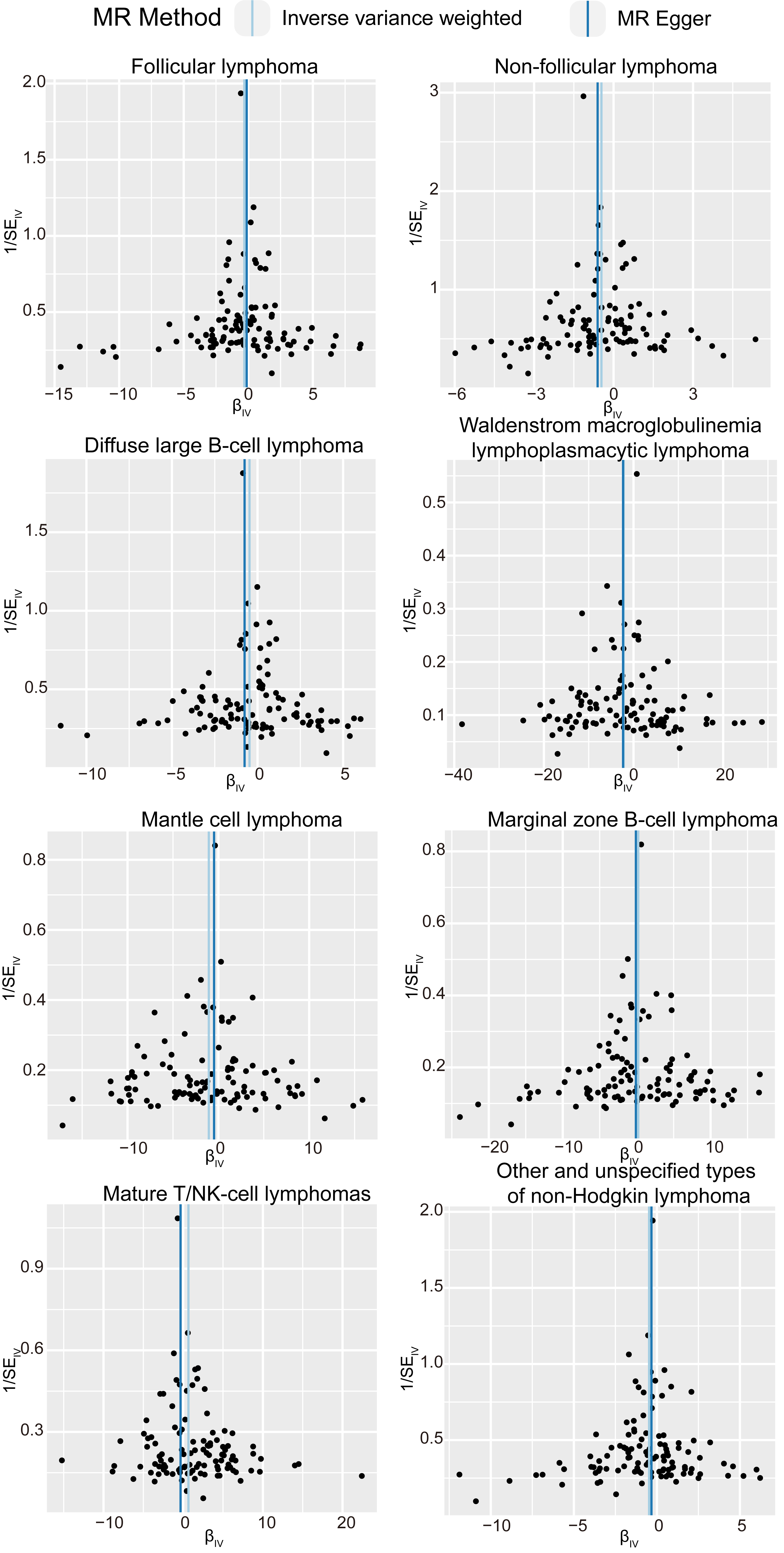

Supplement: Supplemental Figure 8 — Funnel plot for TL displays the estimation obtained through the utilization of the inverse of the standard error of the causal estimate, with each individual SNP serving as a tool. [file crc-24-0402_supplemental_figure_8_suppsf8.jpeg]
