# Supplementary material for: Causality between Telomere Length and the Risk of Hematologic Malignancies: A Bidirectional Mendelian Randomization Study
Source: Cancer Res Commun. 2024 Oct 28;4(10):2815–22. doi: 10.1158/2767-9764.CRC-24-0402 (PMC11513617; doi:10.1158/2767-9764.CRC-24-0402)
Supplement: Supplemental Table 1 — GWAS data information of TL and hematologic malignancies [file crc-24-0402_supplemental_table_1_suppst1.docx]

**Supplemental Table 1. GWAS data information of TL and hematologic malignancies**

| **Name** | **Sample size** | **nCase** | **nControl** | **Sex** | **Population** | **Number of SNPs** |
| --- | --- | --- | --- | --- | --- | --- |
| Telomere length | 472174 | / | / | Males and Females | European | 20134421 |
| Primary lymphoid and hematopoietic malignant neoplasms | 307867 | 7915 | 299952 |  |  | 20168342 |
| Acute myeloid leukemia | 287367 | 231 | 287136 |  |  | 20167489 |
| Chronic myeloid leukemia | 375390 | 232 | 375158 |  |  | 20167476 |
| Acute lymphocytic leukemia | 287320 | 184 | 287136 |  |  | 20167484 |
| Chronic lymphocytic leukemia | 287757 | 624 | 287133 |  |  | 20167519 |
| Leukemia of unspecified cell type | 300172 | 220 | 299952 |  |  | 20168031 |
| Multiple myeloma | 301201 | 1249 | 299952 |  |  | 20167514 |
| Non-Hodgkin lymphoma | 288065 | 928 | 287137 |  |  | 20167533 |
| Hodgkin lymphoma | 300732 | 780 | 299952 |  |  | 20168053 |
| Follicular lymphoma | 301033 | 1081 | 299952 |  |  | 20168074 |
| Non-follicular lymphoma | 302554 | 2602 | 299952 |  |  | 20168109 |
| Diffuse large B-cell lymphoma | 288147 | 1010 | 287137 |  |  | 20167520 |
| Waldenstrom macroglobulinemia, lymphoplasmacytic lymphoma | 287224 | 87 | 287137 |  |  | 20167467 |
| Mantle cell lymphoma | 287336 | 199 | 287137 |  |  | 20167483 |
| Marginal zone B-cell lymphoma | 287329 | 192 | 287137 |  |  | 20167487 |
| Mature T/NK-cell lymphomas | 300287 | 335 | 299952 |  |  | 20168041 |
| Other and unspecified types of non-Hodgkin lymphoma | 301040 | 1088 | 299952 |  |  | 20168070 |
| Leukemia | 373276 | 1260 | 372016 |  |  | 9880879 |
| Lymphoid leukemia | 372776 | 760 | 372016 |  |  | 9015063 |
| Myeloid leukemia | 372478 | 462 | 372016 |  |  | 8171258 |
