# Supplementary material for: Causality between Telomere Length and the Risk of Hematologic Malignancies: A Bidirectional Mendelian Randomization Study
Source: Cancer Res Commun. 2024 Oct 28;4(10):2815–22. doi: 10.1158/2767-9764.CRC-24-0402 (PMC11513617; doi:10.1158/2767-9764.CRC-24-0402)
Supplement: Supplemental Table 2 — The reverse MR analysis on telomere length and hematologic malignancies [file crc-24-0402_supplemental_table_2_suppst2.docx]

**Supplemental Table 2. The reverse MR analysis on telomere length and hematologic malignancies**

| **Exposure** | **nSNP** | **Methods** | **P value** | **OR** | **CI (95%)** | **P(*heterogeneity*)** | | **P(*pleiotropy*)** | **MR**-**PRESSO** |
| --- | --- | --- | --- | --- | --- | --- | --- | --- | --- |
|  |  |  |  |  |  | **MR Egger** | **IVW** |  |  |
| Primary lymphoid and hematopoietic malignant neoplasms |  | MR Egger | 0.626 | 1.005 | 0.986-1.023 | 0.104 | 0.111 | 0.441 | 0.103 |
|  | 29 | Weighted median | 0.240 | 0.992 | 0.980-1.005 |  |  |  |  |
|  |  | IVW | 0.725 | 0.998 | 0.989-1.008 |  |  |  |  |
| Acute myeloid leukemia | 9 | MR Egger | 0.694 | 1.002 | 0.994-1.009 | 0.217 | 0.279 | 0.669 | 0.353 |
|  |  | Weighted median | 0.497 | 0.998 | 0.994-1.003 |  |  |  |  |
|  |  | IVW | 0.943 | 1.000 | 0.996-1.004 |  |  |  |  |
| Chronic myeloid leukemia | 4 | MR Egger | 0.367 | 1.011 | 0.992-1.031 | 0.422 | 0.503 | 0.513 | 0.532 |
|  |  | Weighted median | 0.498 | 1.003 | 0.995-1.011 |  |  |  |  |
|  |  | IVW | 0.241 | 1.004 | 0.997-1.011 |  |  |  |  |
| Acute lymphocytic leukemia | 6 | MR Egger | 0.994 | 1.000 | 0.986-1.014 | 0.385 | 0.527 | 0.993 | 0.531 |
|  |  | Weighted median | 0.708 | 1.001 | 0.996-1.007 |  |  |  |  |
|  |  | IVW | 0.995 | 1.000 | 0.996-1.004 |  |  |  |  |
| Chronic lymphocytic leukemia | 36 | MR Egger | 0.915 | 1.000 | 0.993-1.008 | 0.075 | 0.093 | 0.997 | 0.098 |
|  |  | Weighted median | 0.395 | 1.002 | 0.998-1.005 |  |  |  |  |
|  |  | IVW | 0.782 | 1.000 | 0.998-1.003 |  |  |  |  |
| Leukemia of unspecified cell type | 4 | MR Egger | 0.624 | 0.996 | 0.983-1.010 | 0.518 | 0.618 | 0.564 | 0.697 |
|  |  | Weighted median | 0.567 | 1.002 | 0.996-1.008 |  |  |  |  |
|  |  | IVW | 0.857 | 1.000 | 0.996-1.005 |  |  |  |  |
| Multiple myeloma | 15 | MR Egger | 0.519 | 1.002 | 0.997-1.006 | 0.784 | 0.727 | 0.226 | 0.758 |
|  |  | Weighted median | 0.948 | 1.000 | 0.996-1.004 |  |  |  |  |
|  |  | IVW | 0.674 | 0.999 | 0.996-1.002 |  |  |  |  |
| Hodgkin lymphoma | 14 | MR Egger | 0.886 | 1.001 | 0.992-1.009 | 0.688 | 0.721 | 0.500 | 0.756 |
|  |  | Weighted median | 0.255 | 1.003 | 0.998-1.009 |  |  |  |  |
|  |  | IVW | 0.171 | 1.003 | 0.999-1.007 |  |  |  |  |
| Non-Hodgkin lymphoma | 15 | MR Egger | 0.915 | 0.999 | 0.989-1.010 | 0.102 | 0.126 | 0.625 | 0.162 |
|  |  | Weighted median | 0.493 | 1.002 | 0.996-1.009 |  |  |  |  |
|  |  | IVW | 0.526 | 1.002 | 0.996-1.007 |  |  |  |  |

Abbreviations: SNP, single nucleotide polymorphisms; IVW, inverse variance weighted; OR, odds ratio; CI, confidence interval.
