# Supplementary material for: Causality between Telomere Length and the Risk of Hematologic Malignancies: A Bidirectional Mendelian Randomization Study
Source: Cancer Res Commun. 2024 Oct 28;4(10):2815–22. doi: 10.1158/2767-9764.CRC-24-0402 (PMC11513617; doi:10.1158/2767-9764.CRC-24-0402)
Supplement: Supplemental Table 3 — The reverse MR analysis on telomere length and the classification non-Hodgkin lymphoma [file crc-24-0402_supplemental_table_3_suppst3.docx]

**Supplemental Table 3. The reverse MR analysis on telomere length and the classification non-Hodgkin lymphoma**

| **Outcome** | **nSNP** | **Methods** | **P value** | **OR** | **CI (95%)** | **P(*heterogeneity*)** | | **P(*pleiotropy*)** | **MR**-**PRESSO** |
| --- | --- | --- | --- | --- | --- | --- | --- | --- | --- |
|  |  |  |  |  |  | **MR Egger** | **IVW** |  |  |
| Follicular lymphoma | 24 | MR Egger | 0.473 | 1.002 | 0.996-1.009 | 0.645 | 0.664 | 0.437 | 0.668 |
|  |  | Weighted median | 0.688 | 1.001 | 0.996-1.006 |  |  |  |  |
|  |  | IVW | 0.929 | 1.000 | 0.997-1.003 |  |  |  |  |
| Non-follicular lymphoma | 19 | MR Egger | 0.122 | 1.011 | 0.998-1.023 | 0.589 | 0.406 | 0.072 | 0.424 |
|  |  | Weighted median | 0.675 | 1.002 | 0.993-1.011 |  |  |  |  |
|  |  | IVW | 0.977 | 1.000 | 0.993-1.007 |  |  |  |  |
| Diffuse large B-cell lymphoma | 11 | MR Egger | 0.771 | 1.002 | 0.988-1.016 | 0.611 | 0.681 | 0.660 | 0.698 |
|  |  | Weighted median | 0.660 | 1.002 | 0.994-1.009 |  |  |  |  |
|  |  | IVW | 0.788 | 0.999 | 0.993-1.005 |  |  |  |  |
| Waldenstrom macroglobulinemia, lymphoplasmacytic lymphoma | 0 | MR Egger |  |  |  | / | / | / | / |
|  |  | Weighted median | / | / | / |  |  |  |  |
|  |  | IVW |  |  |  |  |  |  |  |
| Mantle cell lymphoma | 5 | MR Egger | 0.731 | 1.001 | 0.995-1.008 | 0.669 | 0.660 | 0.422 | 0.640 |
|  |  | Weighted median | 0.889 | 1.000 | 0.995-1.005 |  |  |  |  |
|  |  | IVW | 0.586 | 0.999 | 0.995-1.003 |  |  |  |  |
| Marginal zone B-cell lymphoma | 4 | MR Egger | 0.528 | 0.993 | 0.976-1.011 | 0.786 | 0.895 | 0.756 | 0.882 |
|  |  | Weighted median | 0.309 | 0.997 | 0.992-1.003 |  |  |  |  |
|  |  | IVW | 0.092 | 0.996 | 0.992-1.001 |  |  |  |  |
| Mature T/NK-cell lymphomas | 5 | MR Egger | 0.467 | 0.986 | 0.953-1.020 | 0.532 | 0.573 | 0.461 | 0.676 |
|  |  | Weighted median | 0.513 | 1.002 | 0.996-1.009 |  |  |  |  |
|  |  | IVW | 0.990 | 1.000 | 0.994-1.006 |  |  |  |  |
| Other and unspecified types of non-Hodgkin lymphoma | 14 | MR Egger | 0.375 | 0.996 | 0.989-1.004 | 0.356 | 0.425 | 0.741 | 0.466 |
|  |  | Weighted median | 0.236 | 0.996 | 0.990-1.002 |  |  |  |  |
|  |  | IVW | 0.264 | 0.997 | 0.993-1.002 |  |  |  |  |

Abbreviations: SNP, single nucleotide polymorphisms; IVW, inverse variance weighted; OR, odds ratio; CI, confidence interval.
