# Supplementary material for: Causality between Telomere Length and the Risk of Hematologic Malignancies: A Bidirectional Mendelian Randomization Study
Source: Cancer Res Commun. 2024 Oct 28;4(10):2815–22. doi: 10.1158/2767-9764.CRC-24-0402 (PMC11513617; doi:10.1158/2767-9764.CRC-24-0402)
Supplement: Supplemental Table 4 — The Replicated MR analysis on telomere length and hematologic malignancies [file crc-24-0402_supplemental_table_4_suppst4.docx]

**Supplemental Table 4. The Replicated MR analysis on telomere length and hematologic malignancies**

| **Outcome** | **nSNP** | **Methods** | **P value** | **OR** | **CI (95%)** | **P(*heterogeneity*)** | | **P(*pleiotropy*)** | **MR**-**PRESSO** | **Reverse MR** | |
| --- | --- | --- | --- | --- | --- | --- | --- | --- | --- | --- | --- |
|  |  |  |  |  |  | **MR Egger** | **IVW** |  |  | **P value** | **OR** |
| Leukemia | 150 | MR Egger | **4.04E-05** | 1.004 | 1.002-1.006 | 0.030 | 0.021 | 0.061 | 0.017 | 0.801 | 0.862 |
|  |  | Weighted median | **3.64E-05** | 1.004 | 1.002-1.005 |  |  |  |  |  |  |
|  |  | IVW | **2.35E-06** | 1.003 | 1.002-1.004 |  |  |  |  |  |  |
| Lymphoid leukemia | 143 | MR Egger | **5.04E-05** | 1.003 | 1.002-1.005 | 0.348 | 0.336 | 0.219 | 0.348 | 0.094 | 14.727 |
|  |  | Weighted median | **3.73E-06** | 1.003 | 1.002-1.004 |  |  |  |  |  |  |
|  |  | IVW | **1.45E-08** | 1.002 | 1.002-1.003 |  |  |  |  |  |  |
| Myeloid leukemia | 135 | MR Egger | **0.026** | 1.001 | 1.000-1.003 | 0.377 | 0.308 | 0.046 | 0.288 | 0.744 | 24.672 |
|  |  | Weighted median | 0.209 | 1.001 | 1.000-1.002 |  |  |  |  |  |  |
|  |  | IVW | 0.316 | 1.000 | 1.000-1.001 |  |  |  |  |  |  |

Abbreviations: SNP, single nucleotide polymorphisms; IVW, inverse variance weighted; OR, odds ratio; CI, confidence interval.
