# Supplementary material for: Causality between Telomere Length and the Risk of Hematologic Malignancies: A Bidirectional Mendelian Randomization Study
Source: Cancer Res Commun. 2024 Oct 28;4(10):2815–22. doi: 10.1158/2767-9764.CRC-24-0402 (PMC11513617; doi:10.1158/2767-9764.CRC-24-0402)
Supplement: Supplemental Figure Legend [file crc-24-0402_supplemental_figure_legend_suppsfl.docx]

**Supplemental Figure Legend**

**Supplemental Figure 1. Scatter plot of single nucleotide polymorphism potential effects on TL and hematopoietic malignancies.** The vertical lines represent the 95% CI for the effect size on TL, whereas the horizontal lines indicate the 95% CI for the effect size on hematopoietic malignancies. The slope of the fitted lines portrays the estimated MR effect for each method. A, Primary lymphoid and hematopoietic malignant neoplasms; B, Acute myeloid leukemia; C, Acute lymphocytic leukemia; D, Chronic myeloid leukemia; E, Chronic lymphocytic leukemia; F, Leukemia of unspecified cell type; G, Multiple myeloma; H, Hodgkin lymphoma; I, non-Hodgkin lymphoma.

**Supplemental Figure 2. Forest plot of the results of the leave-one-out analysis for forward MR analysis between TL and hematologic malignancies.** The results remained stable, regardless of the removal of any single SNP. A, Primary lymphoid and hematopoietic malignant neoplasms; B, Chronic myeloid leukemia; C, Chronic lymphocytic leukemia; D, Leukemia of unspecified cell type; E, Multiple myeloma; F, Hodgkin lymphoma; G, Non-Hodgkin lymphoma.

**Supplemental Figure 3. Funnel plot for TL displays the estimation obtained through the utilization of the inverse of the standard error of the causal estimate, with each individual SNP serving as a tool.** A, Primary lymphoid and hematopoietic malignant neoplasms; B, Acute myeloid leukemia; C, Acute lymphocytic leukemia; D, Chronic myeloid leukemia; E, Chronic lymphocytic leukemia; F, Leukemia of unspecified cell type; G, Multiple myeloma; H, Hodgkin lymphoma; I, non-Hodgkin lymphoma.

**Supplemental Figure 4. Scatter plot of single nucleotide polymorphism potential effects on hematopoietic malignancies and TL.** The vertical lines represent the 95% CI for the effect size on hematopoietic malignancies, whereas the horizontal lines indicate the 95% CI for the effect size on TL. The slope of the fitted lines portrays the estimated MR effect for each method. A, Primary lymphoid and hematopoietic malignant neoplasms; B, Acute myeloid leukemia; C, Acute lymphocytic leukemia; D, Chronic myeloid leukemia; E, Chronic lymphocytic leukemia; F, Leukemia of unspecified cell type; G, Multiple myeloma; H, Hodgkin lymphoma; I, Non-Hodgkin lymphoma.

**Supplemental Figure 5. Scatter plot of single nucleotide polymorphism potential effects on TL and non-Hodgkin lymphoma.** The vertical lines represent the 95% CI for the effect size on TL, whereas the horizontal lines indicate the 95% CI for the effect size on non-Hodgkin lymphoma. The slope of the fitted lines portrays the estimated MR effect for each method. A, Follicular lymphoma; B, Non-follicular lymphoma; C, Diffuse large B-cell lymphoma; D, lymphoplasmacytic lymphoma (Waldenstrom macroglobulinemia); E, Mantle cell lymphoma; F, Marginal zone B-cell lymphoma; G, Mature T/NK-cell lymphomas; H, Other and unspecified types of non-Hodgkin lymphoma.

**Supplemental Figure 6. Scatter plot of single nucleotide polymorphism potential effects on non-Hodgkin lymphoma and TL.** The vertical lines represent the 95% CI for the effect size on non-Hodgkin lymphoma, whereas the horizontal lines indicate the 95% CI for the effect size on TL. The slope of the fitted lines portrays the estimated MR effect for each method. A, Follicular lymphoma; B, Non-follicular lymphoma; C, Diffuse large B-cell lymphoma; D, Mantle cell lymphoma; E, Marginal zone B-cell lymphoma; F, Mature T/NK-cell lymphomas; G, Other and unspecified types of non-Hodgkin lymphoma.

**Supplemental Figure 7. Forest plot of the results of the leave-one-out analysis for forward MR analysis between TL and non-Hodgkin lymphoma.** The results remained stable, regardless of the removal of any single SNP. A, Non-follicular lymphoma; B, Diffuse large B-cell lymphoma; C, lymphoplasmacytic lymphoma (Waldenstrom macroglobulinemia); D, Mantle cell lymphoma; E, Other and unspecified types of non-Hodgkin lymphoma.

**Supplemental Figure 8. Funnel plot for TL displays the estimation obtained through the utilization of the inverse of the standard error of the causal estimate, with each individual SNP serving as a tool.** A, Follicular lymphoma; B, Non-follicular lymphoma; C, Diffuse large B-cell lymphoma; D, Mantle cell lymphoma; E, Marginal zone B-cell lymphoma; F, Mature T/NK-cell lymphomas; G, Other and unspecified types of non-Hodgkin lymphoma.
